# Tafazzin Mutation Affecting Cardiolipin Leads to Increased Mitochondrial Superoxide Anions and Mitophagy Inhibition in Barth Syndrome

**DOI:** 10.3390/cells9102333

**Published:** 2020-10-21

**Authors:** Patrice X. Petit, Hector Ardilla-Osorio, Lucile Penalvia, Rainey Nathan E.

**Affiliations:** 1 SSPIN Saints-Pères Paris Institut de Neurosciences, CNRS UMR 8003, “Mitochondria, Apoptosis and Autophagy Signalling” Université de Paris—Campus Saint-Germain, 45 rue des Saints-Pères, 75006 Paris, France; lucillepenalva@gmail.com (L.P.); nathan.rainey@gmail.com (R.N.E.); 2 Laboratoire Cellules Souches et Prions, INSERM-S 1124, Université de Paris—Campus Saint-Germain, 45 rue des Saints Pères, 75006 Paris, France; hector.ardila-osorio@parisdescartes.fr

**Keywords:** Barth syndrome, mitochondria, cardiolipin, autophagy, apoptosis, electron transport, tafazzin

## Abstract

Tafazzin is a phospholipid transacylase that catalyzes the remodeling of cardiolipin, a mitochondrial phospholipid required for oxidative phosphorylation. Mutations of the tafazzin gene cause Barth syndrome, which is characterized by mitochondrial dysfunction and dilated cardiomyopathy, leading to premature death. However, the molecular mechanisms underlying the cause of mitochondrial dysfunction in Barth syndrome remain poorly understood. We again highlight the fact that the tafazzin deficiency is also linked to defective oxidative phosphorylation associated with oxidative stress. All the mitochondrial events are positioned in a context where mitophagy is a key element in mitochondrial quality control. Here, we investigated the role of tafazzin in mitochondrial homeostasis dysregulation and mitophagy alteration. Using a HeLa cell model of tafazzin deficiency, we show that dysregulation of tafazzin in HeLa cells induces alteration of mitophagy. Our findings provide some additional insights into mitochondrial dysfunction associated with Barth syndrome, but also show that mitophagy inhibition is concomitant with apoptosis dysfunction through the inability of abnormal mitochondrial cardiolipin to assume its role in cytoplasmic signal transduction. Our work raises hope that pharmacological manipulation of the mitophagic pathway together with mitochondrially targeted antioxidants may provide new insights leading to promising treatment for these highly lethal conditions.

## 1. Introduction

The study of monogenic mitochondrial cardiomyopathies has been expected to open research avenues and yield insights into the crucial importance of mitochondria during cardiac development and disease. However, even with monogenic diseases, we still lack the knowledge needed to develop a treatment and new avenues of fundamental research are required.

Barth syndrome (BTHS) is an X-linked inherited disorder due to mutations in the TAZ gene. Patients with a defective TAZ gene present primarily with dilated cardiomyopathy along with a combination of neutropenia, recurrent infections, mouth ulcers, myopathy, exercise intolerance, delayed motor milestones, mild learning disabilities, growth delay, delayed puberty, and aciduria [1,2,3]. It is of great interest to notice that all tissues are more or less affected since the other characteristic of BTHS pathology is the autophagic behavior detected in neutrophils. Clinical manifestation of the disease has been shown to vary within the same family containing the same TAZ mutation, implying that companion genes may contribute to disease severity [4,5].

When BTHS goes undiagnosed, sudden cardiac arrest due to ventricular arrhythmia or bacterial septicemia due to neutropenia leads to a fatal outcome for most children within the first few years of life. Although there is no cure, the disease can be managed with standard heart failure medications (or heart transplantation) or ventricular assist devices to prevent arrhythmia, and prophylactic antibiotic treatment and granulocyte colony-stimulating factor to treat recurrent infections [6]. With improved diagnosis and appropriate medical treatments and constant monitoring of symptoms, the survival rate and future of these individuals are much brighter, although the quality of life and lifespan of patients are still far from the norm. Historically regarded as a cardiac disease, BTHS is now considered a multisystem disorder which may first be seen by many different specialists or general practitioners. There is a need for a deeper understanding of the molecular basis of BTHS to aid in moving toward more effective treatments.

Understanding of the molecular basis of BTHS was advanced when it was determined that the protein encoded by the TAZ gene [7,8] is a monolysocardiolipin (MLCL) transacylase that transfers unsaturated fatty acyl chains from phosphatidyl or phosphoethanolamine to MLCL to produce mature cardiolipin (CL). In fact, CL is normally synthesized in a premature form, which is usually deacylated by a phospholipase to generate MLCL, which is reacylated by tafazzin to end in the mature and fully functional CL [9,10,11]. Clearly, CL has functions other than inner mitochondrial structure maintenance. CL is required for the optimal function of the mitochondrial electron transport chain: This means, for example, maximizing of the activity of the respiratory chain complexes and of the ADP/ATP translocase involved in ATP synthesis [12] and ROS production [13]. Interaction of CL with cytochrome *c* plays a role in electron transfer [14,15], but also when destabilized during early apoptosis, in the induction of the caspase-3 [16], in the maintenance of membrane fluidity and of osmotic stability [17], which play a key role in the opening of the permeability transition pores and in the import of mitochondrial proteins [18,19], which could somehow be linked to mitochondrial biogenesis.

Although quite different from the behavior of mammalian tissues, which have an extensive mitochondrial network, Saccharomyces cerevisiae lacking tafazzin function (Δtaz1 mutant) recapitulates many of the observed defects seen in cells from BTHS patients, including defective assembly of respiratory chain supercomplexes, decreased mitochondrial respiration, and increased ROS production [18,20,21,22]. Tafazzin deletion in yeast also causes changes in energy transformation and the osmotic properties of mitochondria [23,24]. Moreover, a recent study has defined the need for genes that are important in yeast cells that lack TAZ, like the gene coding for Yme1, the mitochondrial quality control protein [25]. In Drosophila, a tafazzin mutation induces mitochondrial dysfunction and motor weakness [26]. Tafazzin knockdown in zebrafish induces bradycardia and retarded cardiac development [27]. Moreover, decreased expression of tafazzin has been associated with heart failure [28]. 

CL abnormalities have been implicated in cardiac dysfunction, and are seen in ischemic heart disease and aging [29]. The mechanisms that lead from abnormal CL biogenesis to cardiomyopathy are not well understood. They include dysregulation or dysfunction of numerous processes that are related to oxidative phosphorylation [20,30,31], fission or fusion [21,32,33], iron–sulfur cluster biogenesis [34], protein import [18,22], apoptosis [35,36,37,38,39,40,41], autophagy [36,42], and transport of protein precursors across the mitochondrial membrane (in general metabolites) [18,23,43,44,45,46,47,48,49]. 

We are pursuing our work with stable plasmid-derived ShRNAi transfected HeLa cell lines that were previously established to study the effect of tafazzin alterations (also cardiolipin non-maturation) on mitochondrial functions and apoptotic processes [37,50,51]. The cells are easy to handle and perfectly fit for the dissection of the apoptotic and autophagic signaling pathways when linked to mitochondria. They circumvent the need for more involved manipulations and the technical problems encountered with fibroblasts and Barth syndrome iPSC-derived cardiomyocytes (iPSC-CM).

Since CLs are key elements of many signal transduction pathways, we were curious to see if, in addition to apoptosis alterations [37,50,51], decrease in CLs and changes in their acyl chain composition modify autophagy and more precisely mitophagic processes.

## 2. Materials and Methods

### 2.1. Cell Culture and Transfections

Cervical carcinoma HeLa cell lines were cultured in DMEM supplemented with 10% FCS and L-glutamine. Transfection of HeLa cells was performed using Lipofectamine 2000 (Invitrogen). ShTaz1, and shWT1 stable HeLa cell lines were generated by transfection with pSUPER/shTaz, or pSUPER/shCont, respectively, and selected in G418. The revertant ShTaz1R cell line was generated by co-transfecting shTaz1 HeLa cells with pLpC vector (for the puromycin resistance gene), and pcDNA3/Taz mutant and stable clones were selected in puromycin [50].

### 2.2. Lipid Analysis

Lipid analysis was performed as described previously [52]. Cells were sonicated for 20 s in PBS, and phospholipids were extracted from the equivalent of 1 mg of protein of the homogenate as follows: After the addition of 3 mL of 1/1 chloroform/methanol (vol/vol), the internal standards. 0.4 nmol of tetramyristoyl-CL and 0.16 nmol of dimyristoylphosphatidyl glycerol (Avanti Polar Lipids, Alabaster, AL, USA) were added. This mixture was shaken vigorously and placed on ice for 15 min, after which it was centrifuged at 1000× *g* for 10 min. The supernatant was transferred to new tubes, and the protein pellet was re-extracted with 3 mL of 2/1 chloroform/methanol (vol/vol). The combined organic layers were evaporated under nitrogen at 45 °C. The residue was dissolved in 150 μL chloroform/methanol/water (50/45/5, vol/vol/vol) containing 0.01% NH_4_OH, and 10 μL of the solution was injected into the HPLC mass spectrometry system (Thermo Electron Corporation, Waltham, MA, USA). The column temperature was maintained at 25 °C. The lipid extract was injected onto a 2.1 Å~250 mm silica column with 5 μm particle diameter (Merck, Fort Kenalworth, NJ, USA). The phospholipids were separated from interfering compounds by a linear gradient between solution B (97:3 chloroform/methanol, vol/vol) and solution A (85/15 methanol/water, vol/vol). Solutions A and B contained 0.1 mL and 0.01 mL of 25% (vol/vol) aqueous ammonia per liter of eluent, respectively. The gradient (0.3 mL/min) was as follows: 0–10 min, 20% A to 100% A; 10–12 min, 100% A; 12–12.1 min, 100% A to 0% A; and 12.1–17 min, equilibration with 0% A. All gradient steps were linear, and the total analysis time, including the equilibration, was 17 min. A splitter between the HPLC column and the mass spectrometer was used, and 75 μL/min eluent was introduced into the mass spectrometer. A TSQ Quantum AM (Thermo Fisher Scientific, Waltham, MA, USA) was used in the negative electrospray ionization mode. Nitrogen was used as nebulizing gas. The source collision-induced dissociation collision energy was set at 10 V, the spray voltage used was 3,600 V, and the capillary temperature was 300 °C. Mass spectra of CL and MLCL molecular species were obtained by continuous scanning from m/z 400 to m/z 1000 with a scan time of 2 s. The spectra of CL and MLCL species were acquired during their corresponding retention time in the HPLC elution profile. The CL internal standard was set at 100% in each spectrum.

### 2.3. Analysis of HeLa Cell Proliferation Using xCELLigence

The in vitro adhesion and proliferation of adherent ShWT1, ShTaz1 and ShTaz1R cells were studied using an xCELLigence RTDP system (ACEA Biosciences, San Diego, CA, USA) [53]. his device is a label-free, real-time cell analysis system that measures the impedance change of an electrode when cells are adhering, spreading, or dying on it. 20,000 cells/well (200 μL) were seeded into E-plates and incubated at 37 °C in 95% humidified air and 5% CO_2_. The E-plates were incubated for a further 90 h), during which the cell index was measured. The cell index (CI) is a dimensionless parameter derived from the relative change in the measured electrical impedance to represent cell status. All experiments were performed in quadruplicate, and the results were averaged. The baseline was realized for reference with DMEM to fill the wells (4 wells per condition).

### 2.4. Cellular Bioenergetic Assays of Mitochondrial Functions

Oxygen consumption rate (OCR) was measured using a Seahorses Biosciences XF24 extracellular flux analyzer and normalized to total protein, determined using the BCA protein assay (Thermo Scientific). 50,000 cells (HeLa cells ShWT1, ShTaz1 and ShTazR1) were seeded in seahorse assay wells in DMEM glutamax (Invitrogen) with 10% FCS. OCR was expressed as pmol/min/10 µg protein. All other functions like basal respiration, F_0_F_1_-ATP synthase, respiration capacity and H^+^ leak are depicted from the Seahorse curves and expressed as pmol/min/10 µg protein. Sequential inhibition of the F_0_F_1_ synthase was performed with oligomycin (1 μM), uncoupling via permeabilization of the inner mitochondrial membrane by mClCCP (0.5 μM) and finally inhibition of the electron transport chain by antimycin (0.5 μM) + rotenone (1 μM).

### 2.5. AMP/ADP/ATP Determination by HPLC and ATP by Luminometry

Adenine nucleotides were separated by HPLC on a C18 column (Polaris 5C18-A, S250*4.6 Repl, Varian, le Plessis-Robinson France. The injection volume was 30 μL. The flow rate was 1 mL/min, and the cartridge was kept at 30 °C in a column oven. The mobile phase was 28 mM pyrophosphate buffer, pH 5.75. These compounds were detected on a spectrophotometer at a wavelength of 254 nm (L4200 UV Detector, Merk, Fort kenalworth, NJ, USA). The retention times of ATP, ADP and AMP were 3, 5, and 9 min, respectively. Chromatograms were integrated with STAR software v.5 (Varian, le Plessis-Robinson, France).

Simple ATP assays were also performed. Cells were grown in the indicated media and ATP assay reagent (Promega) was added directly to wells and light output was measured with a plate luminometer. Readout was normalized to total protein and the results were presented as relative activity (n = 5) or as ATP content as pmoles per μg protein.

### 2.6. Confocal Microscopy

For confocal microscopy, HeLa cells were seeded in culture medium and processed in 4-chamber glass bottom dishes (Cellvis, Mountain View, CA) and analyzed after staining. The cells were stained in suspension in 2 mL tubes as for the flow cytometry, protected from light with constant shaking at 450 rpm. After staining, cells were pelleted and the resulting pellets (5 μM) were mounted on microscope slides using low-melting-point agarose and imaged immediately. All images were acquired with a Leica TCS SP8 laser scanning confocal microscope, equipped with a 63 angström/1.3 NA oil immersion objective and a white light laser. Cells loaded with 4 mM acridine orange (AO) and washed to discard excess dye were excited with the 484 nm laser line, and emission was detected in the 495–551 nm range for green fluorescence and at 585 nm for red fluorescence. Laser power and detector gain were identical for all experiments for a given dye.

### 2.7. Microspectrofluorimetry

The UV-visible confocal laser microspectrofluorometer prototype was built around a Zeiss UMSP 80 UV epifluorescence microscope (Carl Zeiss, Inc., Oberkochen, Germany), optically coupled by UV reflecting mirrors to a Jobin-Yvon HR640 spectrograph (ISA, Longjumeau, France) [54]. The 351 nm UV line of an argon laser (model 2025; Spectra-Physics, Mountain View, CA, USA) was used for either drug (if fluorescent) or fluorochrome excitation. In this case, TMRM for the mitochondrial membrane potential or LysoTracker Green for lysosomal staining. All details have been provided elsewhere [55].

### 2.8. Acridine Orange Staining of the Acidic Compartment

Acridine orange (Molecular Probes, Invitrogen, A-3568) was used in autophagy assays. It crosses lysosomal membranes (and other acidic compartments) and becomes protonated [56]. The protonated dye stacks and stacked AO emits in the red range. If AO is not in an acidic compartment, its emission is in the green range. When taken as a DNA intercalator, for example, its fluorescence intensity decreases when DNA condenses.

As a marker of autophagy, the volume of the cellular acidic compartment was visualized by AO staining [56,57]. Cells were seeded in six-well tissue culture dishes and treated as described above for the cell viability study. Cells were incubated with medium containing 1 μg/mL AO for 15 min. Cells were washed twice with PBS to remove excess AO, fresh medium was added, and fluorescent micrographs were taken using an Olympus inverted fluorescence microscope. All images presented are at the same magnification. The number of cells with increased acidic vesicular organelles was determined by flow cytometry [57]. Cells were trypsinized, harvested and analyzed by BD FACScalibur 4C (using Cellquest software). A minimum of 10,000 cells within the gated region were analyzed. We bore in mind that acidotropic dyes like AO only stain late autophagic vacuoles. 

### 2.9. Determination of Lipid Peroxidation and Protein Carbonylation

We used a lipid peroxidation assay kit (Abcam PLC, Paris, France) to detect malonaldehyde in samples. The free MDA generated during lipid peroxidation refers to the oxidative degradation of lipids and reacts with thiobarbituric acid to generate an MDA-TBA adduct, the absorbance of which was measured at 532 nm. This kit detects levels as low as 1 nmol/well. For the calculation, we determined the MDA concentration in standards and samples from their absorbance as described in the protocol of the lipid peroxidation assay kit (Abcam PLC, Paris, France: ref. ab118970). Protein carbonylation in cell lysates was assayed using Cayman’s Protein Carbonyl Fluorometric Assay Kit. 

### 2.10. Oxidative Stress, Production of Reactive Oxygen Species, and Mitochondrial Membrane Potential

Reactive oxygen species (ROS) generated from the mitochondria of HeLa cells shWT1, shTaz1 and shTaz1R were detected indirectly by quantitatively measuring H_2_O_2_ with the Amplex Red hydrogen peroxide/peroxidase assay kit (Thermo Fisher Scientific, Montigny-le-Bretonneux, France, ref. A22188). Lipid peroxidation products in the HeLa cells were quantified by measuring the level of malondialdehyde using a TBARS kit according to the manufacturer’s instructions (Abcam PLC, Paris, France: ab118970). For other measurements, before staining, cells were trypsinized, washed and resuspended together with their supernatant in the DMEM Glutamax-I culture medium with 10% FSC. The following probes were added to cell samples and incubated for 15 min at 37 °C. For mitochondrial membrane potential determination, 3,3′-dihexyloxacarbocyanine iodide DiOC_6_(3) (Molecular Probes) was used at 20 nM final concentration (stock solution 10 μM). The uncoupling agent carbonylcyanide m-chlorophenylhydrazone (mClCCCP, 1 mM stock solution, 15 min, 37 °C) was added at 10 μM before dye addition and used as a control of ΔΨm drop; for ROS production. 2′,7′-dichlorodihydrofluorescein diacetate (H_2_DCFH-DA, Molecular Probes) was used at 5 μM final concentration for H_2_O_2_ measurements (stock solution at 1 mM) and MitoSOX (Molecular Probes) was used at 5 μM final concentration for superoxide anion (stock solution 1 mM). H_2_O_2_ (5 μM, 30 min) was used as a positive control for the H_2_DCF-DA measurements and 50 μM paraquat was used for superoxide anion generation. Double staining was generally done to assay simultaneously cellular viability, with propidium iodide (PI) at 1 μg/mL final concentration for DiOC_6_(3), H_2_DCFH-DA, and YO-PRO-1, or with TO-PRO-3 iodide at 1 μg/mL final concentration when using MitoSOX staining. 

### 2.11. NAD(P)H and NADH Determination

NAD(P)H fluorescence was elicited with a multiline ultraviolet light set at 400 mW at 360 nm on a FACS Vantage. Changes in the autofluorescence of normal and apoptotic cells were recorded as previously described by Gendron et al. [58]. The light emitted was collected with a 424 ± 40 nm bandpass for NAD(P)H fluorescence. The NAD(P)H content of cells was determined with the NADH/NAD^+^ or the NAD(P)H/NAD(P)^+^ assay Kit (Cell Technology, Inc., Mountain View, CA, USA).

### 2.12. Caspase 3/7 Activation

Isolated HeLa cells were washed and suspended in calcium-free buffer solution (140 mM NaCl, 1.13 mM MgCl_2_, 4.7 mM KCl, 10 mM glucose, 0.1 M EDTA, and 10 mM HEPES, pH 7.2). Cells were then loaded at room temperature for 30 min with fluorescent indicator-linked substrates for activated caspases 3/7 (Caspase-3/7 Green ReadyProbes™ Reagent with a DEVD sequence, Molecular Probes).

All flow cytometry measurements and analyses were done using BD FACS Canto II using BD FACS Diva software. A minimum of 10,000 cells within the FSC/SSC region were recorded and analyzed.

### 2.13. Mitochondrial DNA Assay

Total DNA including mitochondrial DNA was isolated from HeLa cells (ShWT1, ShTaz1, and ShTazR1) using a QIAamp DNA Micro Kit from Qiagen. Mitochondrial NADH dehydrogenase 1 (ND1) gene content was assayed by RT-PCR, as described in semiquantitative and real-time RT-PCR, with 10 ng total DNA as the template using the primers upper 5′-ATGGCCTTCCTCACCCTAGT-3′ and lower 5′-AGAGGGCGTATGG GTTCTTT-3′. ND1 was normalized with genomic gene GAPDH using the same set of primers described in real-time RT-PCR.

### 2.14. Specific Lysosomal Staining

Double staining was used for the analysis of the lysosomal compartment, i.e., an incubation of 20 min at 37 °C as performed with 1 μM LysoTracker Green and TOPRO-3 iodide was added just before measurement to check cell viability [59]. All samples were analyzed by flow cytometry on a FACSCalibur 4C.

### 2.15. Detection of Lysosomes and Autophagolysosomes with Monodansylcadaverine (MDC)

The autofluorescent agent monodansylcadaverine (MDC, Sigma-Aldrich, L’Isle d’Abeau Chesnes, France) was used as a specific autophagolysosome marker. Staining was clearly related to the interaction of the dye with autophagosome membrane lipids [60]. Cells were seeded in six-well tissue culture dishes and treated as described above for the cell viability study. Forty-eight hours following initial treatment, if any, cells were incubated with medium containing 1 μg/mL MDC for 15 min; excess MDC was then removed, cells were washed once with PBS, fresh medium was added, and flow cytometric measurements were performed.

### 2.16. Transmission Electron Microscopy

TEM services, including sample fixation, embedding, ultra-microtomy and staining were provided by the VCU Department of Anatomy and Neurobiology Microscopy Facility. Sections were imaged via a Jeol JEM-1230 transmission electron microscope (EM) equipped with a Gatan UltraScan 4000SP 4K Å~ 4K CCD camera. The magnification of each image is indicated by the scale bar at the bottom of the micrograph.

### 2.17. Immunoblotting

Cells were lysed in modified Laemmli buffer [60 mM Tris (pH 6.8), 10% glycerol (vol/vol), 2% (wt/vol) SDS and bromophenol blue, without 2-mercaptoethanol] by sonication for 30 s on ice and were then centrifuged at 3000× *g* for 5 min. The protein extracts were boiled for 5 min at 100 °C and frozen at −80 °C. Protein concentration was determined by the micro-BCA protein assay (Pierce, Rockford, IL, USA). Cell lysates (20 μg per lane) were resolved by SDS-PAGE (7.5% or 15% (wt/vol) polyacrylamide). Proteins were then electroblotted onto 0.45 μm pore-size nitrocellulose filters, and the filters were blocked by incubation with 5% (wt/vol) non-fat milk in PBS containing 0.1% Tween-20 for 1 h and the filters were blocked by incubation with 5% (wt/vol) non-fat milk in PBS containing 0.1% Tween-20 for 1 h. The filters were then incubated for 1 h at room temperature to determine LC3 cleavage, with rabbit anti-LC3 (ref. L7543 from Sigma-Aldrich, L’Isle d’Abeau Chesnes, France)) at 1:1000 and detected with anti-rabbit, RPN2124 from GE Healthcare, Vélizy-Villacoublay, France) at 1:2000 dilution (catalogue number A2066, Sigma-Aldrich, L’Isle d’Abeau Chesnes, France). Blots were washed for 10 min three times with 0.2% Tween 20 in PBS, and then incubated for 1 h with peroxidase-labeled anti-mouse or anti-rabbit immunoglobulins (at 1:5000). Blots were developed using an enhanced chemiluminescence detection system (ECL2; Amersham, Orsay, France). Rapamycin was used at 50 nM (autophagy inducer) and at 10 nM for bafilomycin A3 as inhibitor.

### 2.18. Semiquantitative and Real-Time RT-PCR

Total RNA isolation from NVMs was performed as described previously (25). RT-PCR was carried out as previously described [61]. Tafazzin mRNA levels were taken as a percentage of control. For real-time PCR, 2 μL of the products of the reverse transcription reaction was amplified using SYBR dye (SA Biosciences, Paris, France) along with the same primers (including tafazzin and GAPDH as normalizer) in a Roche LightCycler V.2 (Roche, Boulogne-Billancourt, France) [61]. Tafazzin mRNA levels were determined using the ΔΔCt method as described by Winer et al. [62] and given as a percentage of control.

### 2.19. 3[H]-Leucine Incorporation Assay

The rate of protein synthesis by the three HeLa cell lines was estimated by 3[H]-leucine incorporation as described by Harding et al. [63]. HeLa cells were placed on six-well plates at a density of one million cells per well. After a 40-h incubation in DMEM containing 10% FBS, the medium was changed to serum-, glucose-, and pvruvate-free DMEM containing 1 μCi 3[H]leucine, and the adenovirus was added. After 48 h, the cells were harvested for trichloroacetic acid precipitates, which were counted for 3H activity (counts/min of [3H] leucine incorporation) in a scintillation counter. 3H activity of HeLa cells infected with a tafazzin shRNA adenovirus represents protein synthesis, expressed as a percentage of control (ShTaz1 and ShTaz1R and ShWT1 with a scrambled adenovirus). 

### 2.20. Transfection and Luciferase Assay

ShWT1, ShTaz1 and ShTaz1R cells were transfected by electropermeabilization using a cuvette with a 2 mm gap (BTX, San Diego, CA, USA) in 0.4 mL PBS buffer containing 0.1% glucose and 1 μg of 1818hBNPluc plasmid DNA per million cells. The cells were placed on 12-well plates and maintained in DMEM containing 10% fetal bovine serum for 40 h. The medium was then changed to serum-, glucose-, and pyruvate-free DMEM; the shRNA adenovirus (100 vp/cell) was added; and the samples were maintained for 48 h. Finally, the cells (ShTaz1 and ShTaz1R) were harvested for luciferase activity [64], which represents human brain natriuretic peptide (hBNP) promoter activity and is expressed as fold increase vs. ShWT1 (cells infected with a scrambled shRNA virus). The 1818hBNPluciferase construct [65] was graciously provided by Dr. M. C. LaPointe (Henry Ford Hospital, Detroit, MI, USA). 

### 2.21. Statistical Analysis

Data are expressed as means ± SE. Differences in mean values were analyzed by two-tailed t-tests or by one-way ANOVA using the Student–Newman–Keuls method for pairwise multiple comparisons. The statistical differences are represented by the *p*-value: * *p* < 0.05, ** *p* < 0.01 and *** *p* < 0.001; a value of *p* < 0.05 was considered significant. The *p* value is cited in the text of the figure when needed.

## 3. Results

### 3.1. Biochemical Features of ShTaz1 and ShTaz1R Cells Versus ShWT1 HeLa Cells

**Figure 1:**
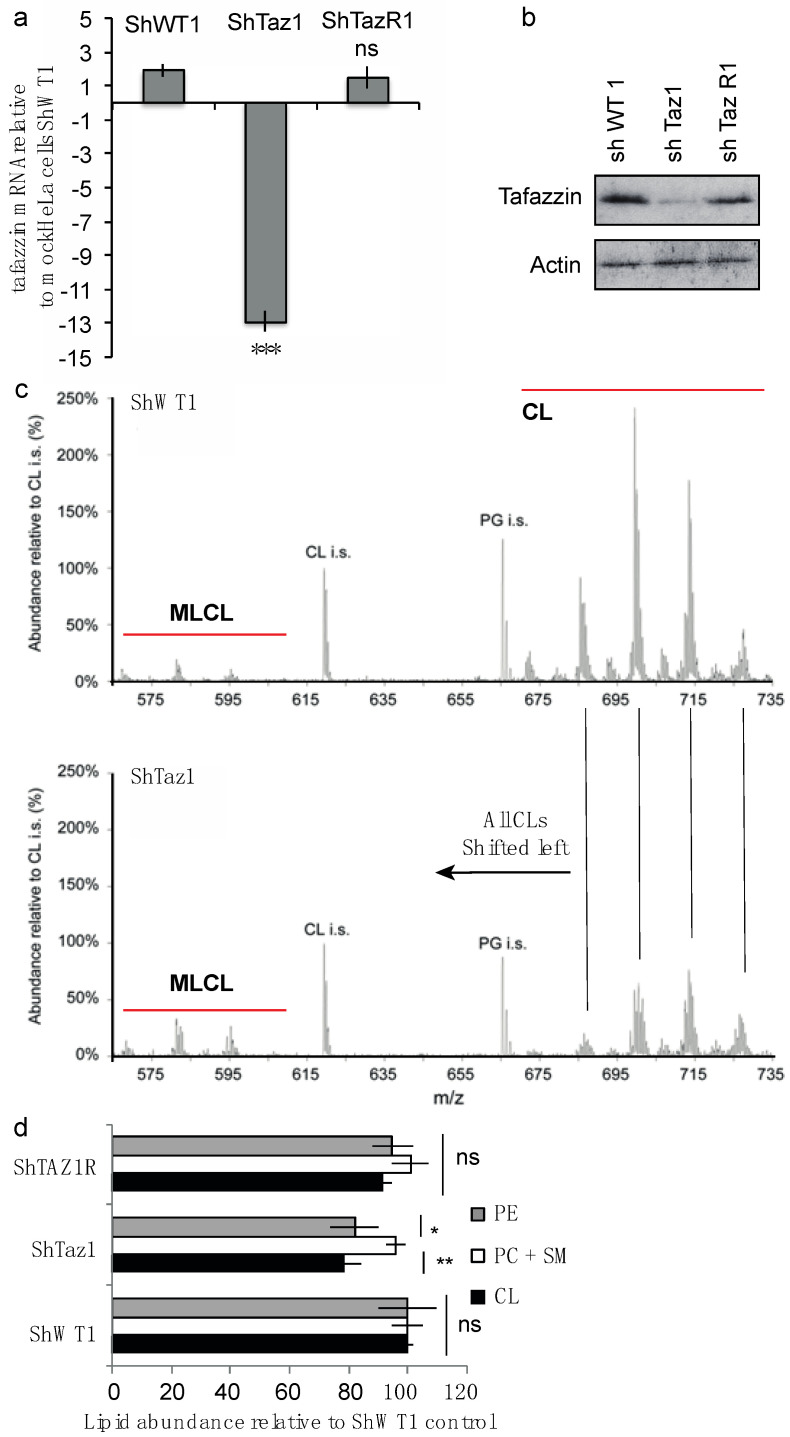
General biochemical characterization of the different cell lines (ShWT1; control, ShTaz1; Tafazzin knockdown and ShTazR1; Tafazzin revertant). (**a**) Measurement of tafazzin mRNA for each cell line (ShWT1, ShTaz1, and ShTazR1) relative to mock HeLa cells as control. Data show mean of 5 independent preparations ± SEM. For comparison one t test was performed. ns = no significant and *** is the *p*-value ≤ 0.001. (**b**) Western blot analysis of tafazzin in the ShWT1, ShTaz1, and ShTazR1 cells. (**c**) Lipid extracts from control and tafazzin knockdown HeLa cells were analyzed for the overall levels of CL, PE and PC together with sphingomyelin (PC + SM). (**d**) Lipid abundance relative to ShWT1 control? For comparison one *t*-test was performed. ns = no significant and * is the *p*-value ≤ 0.05 and ** is the *p*-value ≤ 0.01.

In order to give rise to a cell model for BTHS, the RNA interference strategy was used to knockdown tafazzin. Using either a tafazzin-specific sequence or a nonspecific shRNA control, we constructed short hairpin RNA-encoding plasmids (shRNA), HeLa cells were transfected and stable clones were isolated and characterized [50] (Figure 1a,b)

To eliminate the possibility that Fas resistance could be due to an off-target effect of ShRNA, we introduced a silent mutation into the cDNA of human tafazzin. The ShTaz1 cells transfected with this mutant are a characteristic revertant clone (ShTaz1R). The effective efficiency of the tafazzin-targeting siRNA was then checked by tafazzin mRNA analysis (Figure 1a) and by Western blot (Figure 1b) with ShTaz1R cells expressing tafazzin to a similar level to the endogenous protein. 

#### 3.1.1. Cardiolipin Modifications

Considering the lipid composition of the newly generated tafazzin-deficient cells (ShTaz1), CL abundance was modified as expected (Figure 1c), whereas the other major lipids, i.e., phosphatidylethanolamine (PE), phosphatidylcholine and sphingomyelin, were not significantly affected (Figure 1d). 

Finally, the CL pattern of the HeLa cells knockdown for tafazzin resembles that of the BTHS lymphoblasts [50]. Some differences were due to the modest increase in MLCL measured compared to what was evidenced in BTHS lymphoblastoid cells. The ShWT1 control and the revertant cells (ShTaz1R) exhibited a restored level of CL (Figure 1d).

**Figure 2:**
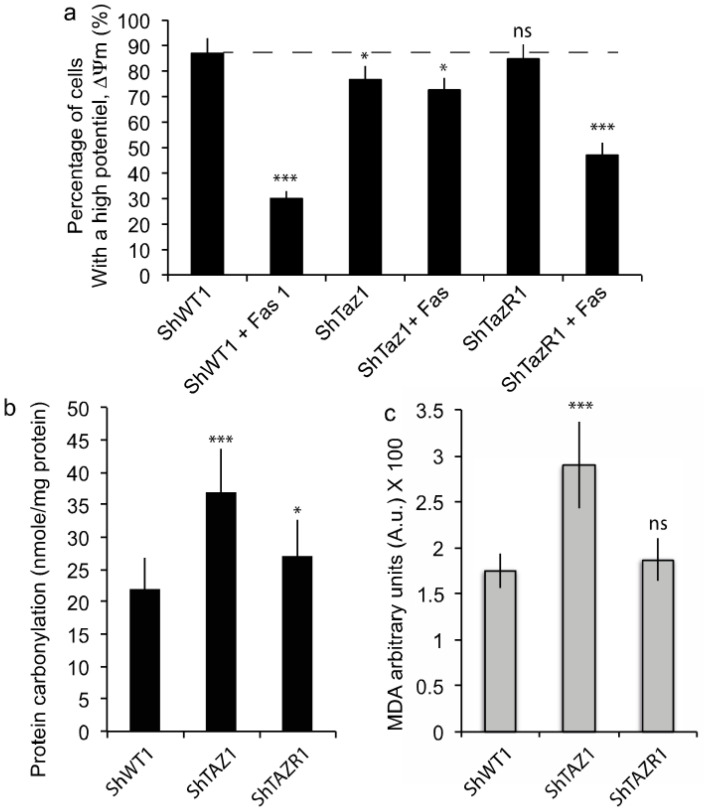
Biochemical characterization of Fas-induced apoptosis and the oxidative cellular context. (**a**) Fas-induced apoptosis is inhibited in ShTaz1 RNAi knockdown cells and the cell death process is restored in the revertant cells (ShTaz1R cells). The ± SEM is related to seven independent preparations. (**b**) Protein carbonylation measurements for ShWT1, ShTaz1 and ShTazR1 cells. (**c**) Malonaldehyde product ion measurements for ShWT1, ShTaz1 and ShTazR1 cells. ns = no significant and * is the *p*-value ≤ 0.05 and *** is the *p*-value ≤ 0.001.

When the different HeLa cell lines (ShWT1, ShTaz1 and ShTaz1R) were treated with 0.5 μg/mL anti-Fas antibody for 24 h it was clear that the tafazzin-deficient cells resisted the induction of Fas apoptosis (Figure 2a), whereas ShWT1 and the revertant cells (ShTaz1R) were sensitive to Fas. These experiments indicate that tafazzin is necessary for efficient Fas-induced apoptosis.

Curiously, the ShTaz1 cells exhibited clear enhancement of their protein carbonylation (Figure 2b) and malonaldehyde (MDA) (Figure 2c), which indicate significant increases in protein oxidation and lipid peroxidation. These events are clearly related to tafazzin depletion, whereas ShWT1 and ShTaz1R did not exhibit such behavior. So, it appears that the growth of ShTaz1 cells occurs in a context of slight elevation of free radical production.

#### 3.1.2. Early Apoptosis-Associated Events are also Inhibited

**Figure 3:**
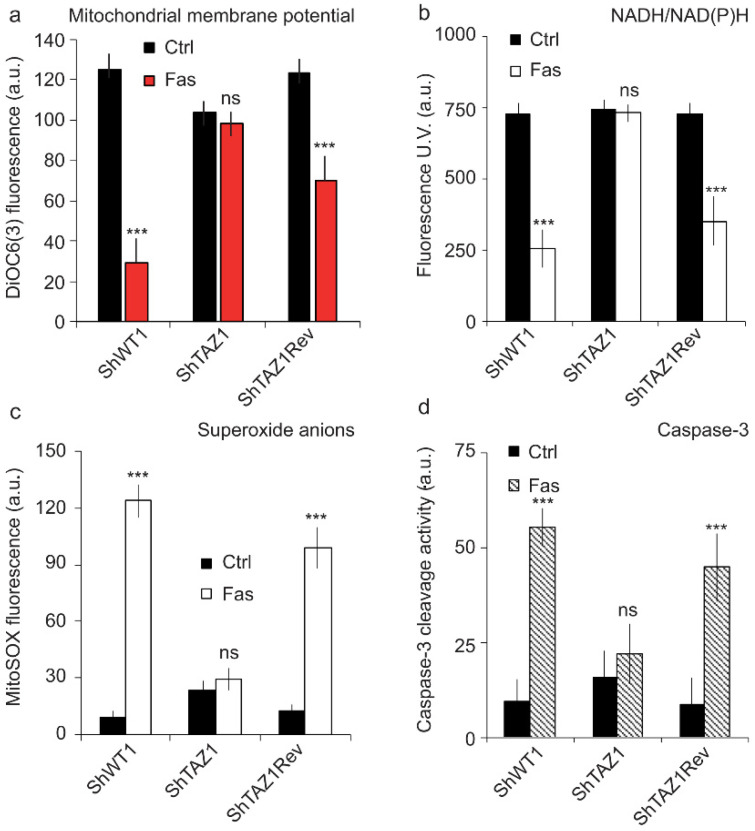
Flow cytometric analysis of the “canonical” early events of apoptosis that are affected in the tafazzin mutant cells (ShThaz1). (**a**) Measurement of the mitochondrial membrane potential (ΔΨm) with DiOC_6_(3) in the three cell lines when treated with Fas. (**b**) Estimation of the NAD(P)H reduction (The fluorescent NADH and NADPH give rise to non-fluorescent NAD^+^ or NADP^+^) in the three cell lines when treated with Fas, which is concomitant with potential changes in the mitochondrial membrane, when they occur. (**c**) Measurement of the superoxide anions produced by the defective mitochondrial electron transport chain during Fas-induced apoptosis. (**d**) Caspase-3 activity associated with cytochrome c release from the mitochondria when Fas-induced apoptosis is occurring. ns = no significant and *** is the *p*-value ≤ 0.001.

Since Fas-induced apoptosis is inhibited in the mutant cells (ShTaz1), we carefully examined all classically associated apoptotic events to see if the associated biochemical events like mitochondrial membrane potential (ΔΨm) drop, (NADH/NAD(P)H reduction, ROS production (superoxide anions) and caspase-3/7 activity were also affected (general cell death profile). So, we measured both the ΔΨm and NADH/NAD(P)H reduction by flow cytometry analysis. ΔΨm and NADH/NAD(P)H are supposed to undergo a huge reduction, respectively, as early events of the apoptotic process [59], but were almost unaffected (Figure 3a,b) in ShTaz1 tafazzin mutants, whereas ShWT1 and ShTaz1R exhibited a drop in ΔΨm and NAD(P)H gave rise to NAD(P)^+^. Except for a small decrease in ΔΨm (Figure 3a), no other changes were seen after Fas induction.

With or without Fas treatment, the tafazzin knockdown cells (ShTaz1) showed small increases of superoxide anions and of caspase-3/7 activity (Figure 3c,d respectively). ROS production and capase-3/7 activity have an effect on long-term cellular behavior, but do not seem to cross a threshold that may promote cell apoptosis.

### 3.2. Cell Adhesion and Proliferation

The strength of cell adhesion is represented as the cell index (CI, unitless measurement, which represents resistivity). As cells adhere to the E-plates, the value of CI increases from zero, and this will usually be evident within the first 10–15 min of seeding. As adhesion ends, there will be a concordant decrease in CI and a transition phase takes place with stabilization of CI before a new increase of CI when cells proliferate. At the end of the proliferation phase, when CI peaks, CI decreases slowly as the cells retract from the E-plate and then detach.

**Figure 4:**
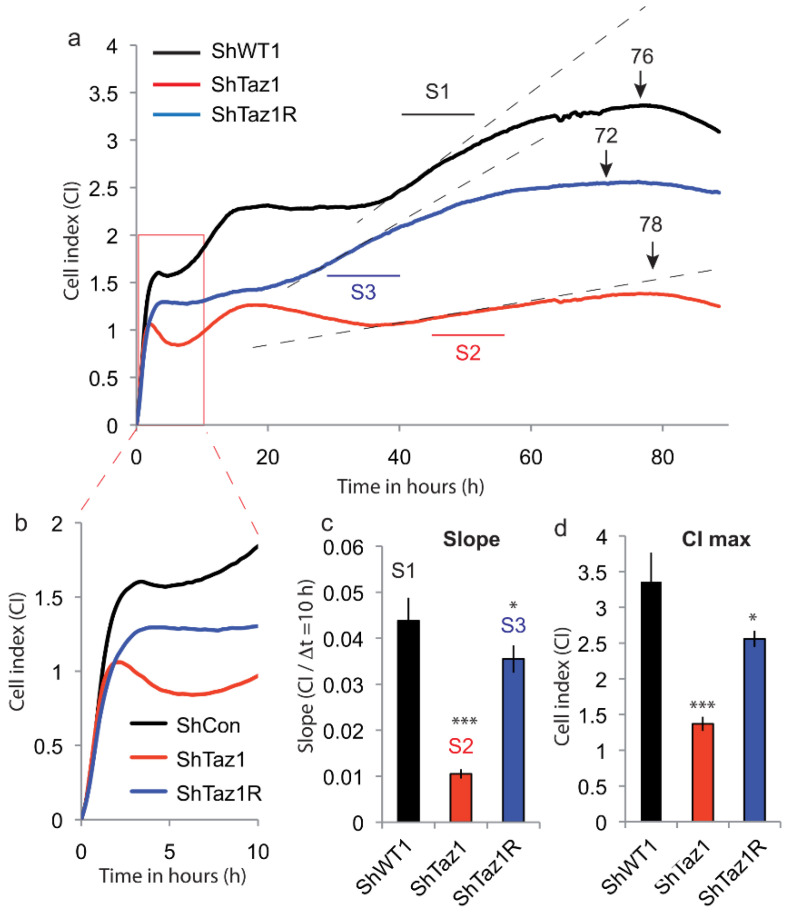
Analysis of the cellular proliferation of the three lines (ShWT1, ShTaz1 and ShTazR1) by impedancemetry with the Xcelligence system (ACEA, Invitrogen). (**a**) The histogram represents the cell index (CI) versus time of proliferation (in h) of the three cell lines ShWT1, ShTaz1 and ShTaz1R. S1 to S3 are the slope measurements over 10 h for each proliferation curve. SWT1 in black, ShTaz1 in red and ShTaz1R in blue. The number on the top of the curve indicates the number of hours needed to reach peak proliferation for each cell line. (**b**) Cellular enlargement and the early phase of cell adhesion to the bottom of the wells for the three cell lines. (**c**) Histogram representation of the slope of cellular proliferation of the three cell lines. The slope is given as CI/Δt (recorded over ten hours between 50 and 60 h of cell culture). (**d**) Histogram representation of the maximum CI reached by the three cell lines at their peak proliferation. CI is in arbitrary units (a.u.). * is the *p*-value ≤ 0.05 and *** is the *p*-value ≤ 0.001.

All these HeLa cells (ShWT1, ShTaz1 and ShTaz1R) were weakly adherent (this is manifested by a quite low CI of 2–2.5), whereas very adherent cells may reach a CI of 10 [66,67] and also proliferate until they form a monolayer (Figure 4). The initial increase (0–1.5 h) in CI is associated with the attachment and adhesion of the cells, followed by spreading and a brief plateau phase, which is sometimes replaced by a slight decrease in CI. Here, the cells had different adhesion capacities that appeared to be linked to the status of tafazzin (Figure 4b). The progressive linear increase in CI within the 24 h after seeding is consistent with the initiation of the proliferation of these cells. In theory, this temporal profile should only be observed with proliferating cells, whereas nonproliferating cells will remain constant until they become nutrient-deprived and compromised.

In the present case, three cell lines presented distinct initial phases, meaning that their metabolism was different (including ATP production). The different cell lines exhibited no sign of cell death, but ShTaz1 (stable tafazzin knockdown) exhibited cellular proliferation which was deficient if compared to ShWT1 (control) and ShTaz1R (revertant) (Figure 4a). These observations were further confirmed by differences in proliferation slopes and by the CI value reached at the peak proliferation point. It is important to notice that the re-introduction of the TAZ gene in revertant cells (ShTaz1R) does not fully restore the proliferation slope or the high CI value of the control cells (ShWT1) (Figure 4a). The differences in proliferating slope and CI of the proliferation curves are quite characteristic, the ShTaz1 cells having a very low proliferation rate (Figure 4), and a low CI, whereas ShWT1 and ShTAZ1R have higher slopes and CI (Figure 4c,d). It should be noticed also that the proliferation capacity and CI are not totally restored when tafazzin is reintroduced into ShTaz1 cells (ShTaz1R).

### 3.3. Cellular Bioenergetics

#### 3.3.1. Electron Microscopy of the ShTaz1 Tafazzin Mutant

**Figure 5:**
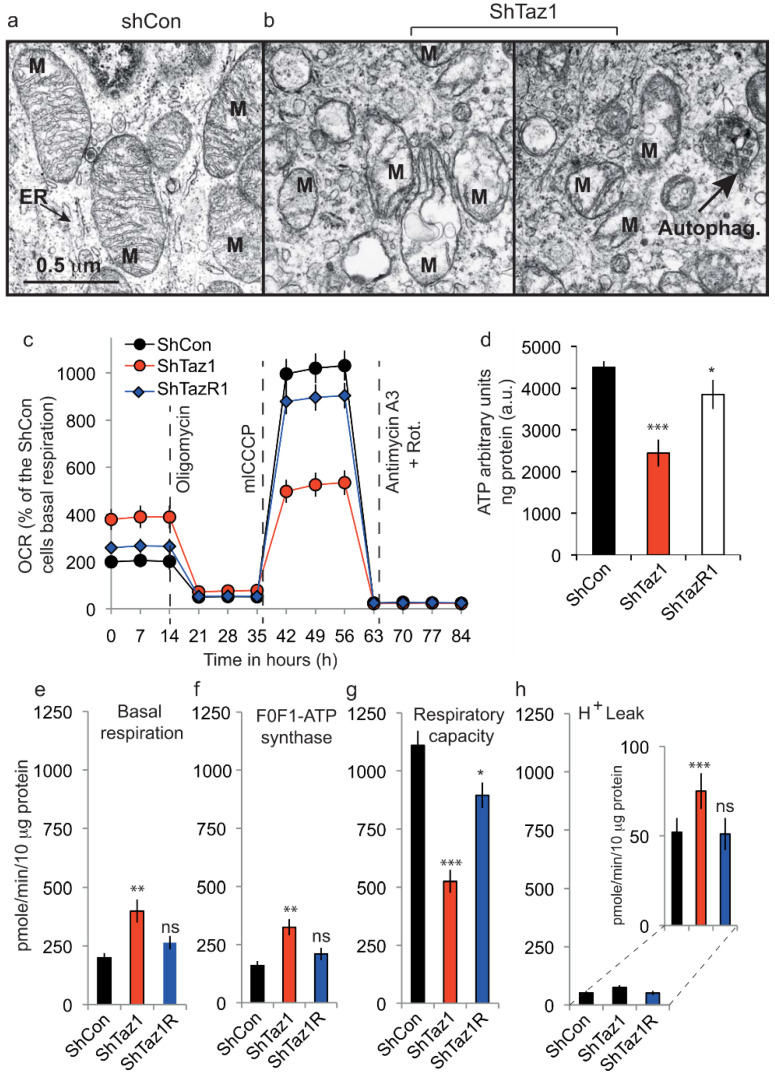
Quantitation of mitochondrial functional parameters; electron microscopy and respiratory activity. (**a**,**b**) Electron microscopy of the ShWT1 and ShTaz1 cells. Left panel, ShWT1 (**a**) and ShTaz1 (**b**, the middle and right panel). In b, two electron microscopy pictures of the different types of mitochondria found for the ShTaz1 cells (middle and left panel). ER; Endoplasmic reticulum, M: Mitochondria; Autoph (indicated by the black arrow), autophagosomes enclosing part of the cytoplasm. (**c**) Analysis of the mitochondrial oxygen consumption rate (OCR) for the three cell lines in response to successive treatments with oligomycin (Oligo, 0.5 μM, an ATP synthase inhibitor), mClCCP (an uncoupler, 0.5 μM) and antimycin + rotenone (Rot) (0.5 μM, complex III inhibitor and for Rot 1 μM) with a Seahorse device XF24 flux analyzer. (**e**) Deduced by calculations from (**d**) Histogram representation of basal respiration. (**f**) Deduced by calculations from (**d**) Histogram representation of F_0_F_1_ATP synthase activity. (**g**) Deduced by calculations from (**d**) Histogram representation of maximal respiration capacity (**h**) Deduced by calculations from (**d**) Histogram representation of the proton leak (H^+^ leak). Since the H^+^ leak is small, there is an enlarged representation on another scale on the left of the primary histogram. In (**e**–**h**), the activities are always measured in pmol/min/10 μg protein. ns = no significant and * is the *p*-value ≤ 0.05 and ** is the *p*-value ≤ 0.01 and *** is the *p*-value ≤ 0.001.

We used electron microscopy to compare the mitochondria of ShWT1 cells and tafazzin-deficient cells (Figure 5a,b). The mitochondria from HeLa cells, even if not so numerous as in mouse hepatocytes, had a classic conformation with two distinct membranes (outer and inner) enclosing a dense matrix with distinct cristae membranes that appeared as stacks perpendicular to the main extension of the mitochondria. A clearly defined endoplasmic reticulum with a lumen was also visible on the micrographs.

In ShTaz1 cells knockdown for tafazzin, the mitochondria were smaller with few cristae membranes and a translucid matrix (Figure 5b, center and left). Some autophagosomes (Autophag.) appeared to be visible on the electron micrograph (Figure 5b, image at the right). The mitochondria were much smaller and dispersed in the cytoplasm with an endoplasmic reticulum that is difficult to identify.

#### 3.3.2. Cellular Light Scattering Properties and Confocal Images Revealed ShTaz1 Cell Hypertrophy

**Figure 6:**
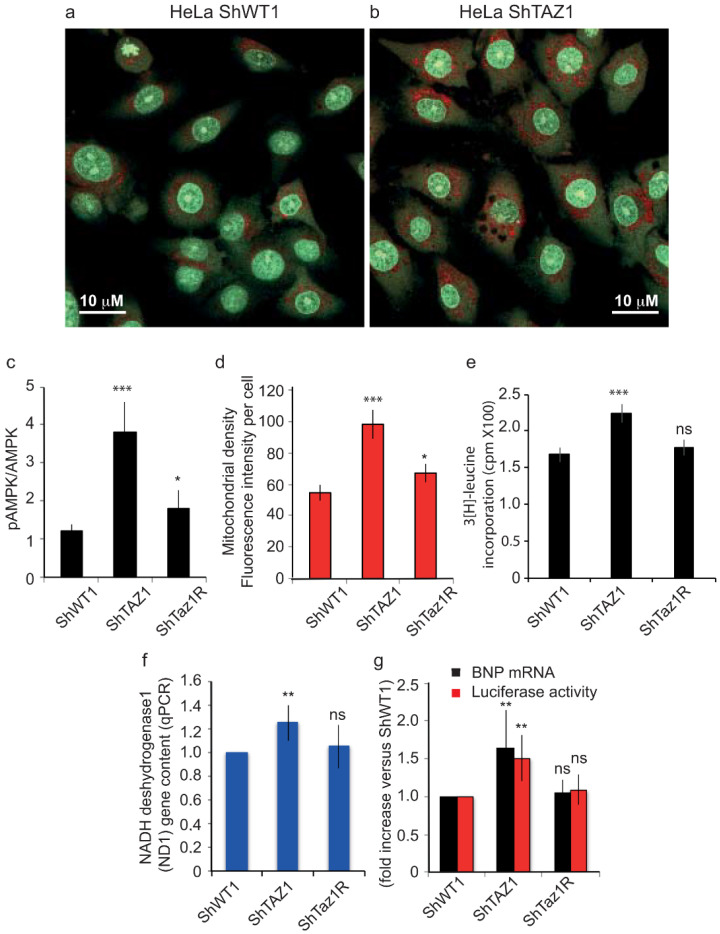
Tafazzin knockdown induces cellular hypertrophy and mitochondrial biogenesis. (**a**) and (**b**) Confocal images of the ShWT1 and ShTaz1 HeLa cells after staining with 5 μM AO as described in materials and methods. AO stains the nuclei (binds to DNA) and part of the cytoplasm in green (non-aggregated form) and also the acidic vesicles where it accumulates and because of its stacking is subject to a Stokes shift and is re-emitted in red (i.e., mainly the lysosomes and the autophagic vacuoles when present). * *p* < 0.05 vs. Control HeLa cells (ShWT1). (**c**) ShTaz1 knockdown cells as well as their revertant ShTAZR1 were treated and assayed for phospho-AMPK (p-AMPK) as described in materials and methods. p-AMPK was corrected for total AMPK and expressed as fold increase vs. ShWT1 control (arbitrarily set at 1). (**d**) Quantification of red fluorescence from TMRM staining on confocal images where the intensity per cell is taken into account. (**e**) ShTaz1 cells exhibited increased protein synthesis. HeLa cells were transduced with the shRNA adenovirus and labeled with 3[H]leucine as described in materials and methods. Protein synthesis is expressed as counts per minute (cpm) of 3[H]leucine incorporated. Data represent means ± SE from 4 separate experiments. From (**c**) to (**g**) the ± SEM is given on 6 independent preparations (**f**) Mitochondrial DNA content as assayed by NADH dehydrogenase 1 (ND1) gene content measured by RT-PCR (see materials and methods). (**g**) Increases expression of the hypertrophic marker brain natriuretic peptide (BNP). RT-PCR is used to detect the BNP mRNA. The BNP promoter was activated in tafazzin knockdown HeLa cells (ShWT1). ShWT1 cells were transfected with a luciferase reporter gene under the control of a BNP promoter and infected with shRNA adenovirus. The BNP promoter activity is detected by luciferase activity, expressed as fold increase vs. SCR control. ns = no significant and * is the *p*-value ≤ 0.05 and ** is the *p*-value ≤ 0.01 and *** is the *p*-value ≤ 0.001.

Before starting the respiratory activity measurements, we examined the three cell lines (ShWT1, ShTaz1 and ShTaz1R) by light microscopy or confocal microscopy. ShTaz1 cells appeared to be slightly swollen and bigger than ShWT1 and ShTAZ1R cells. We used the light scattering capacities of the flow cytometer to check the viability and the biochemical characteristics of the different cell lines. Counterintuitively, the cells that were partly swollen had a less “rough” intrinsic content in side scatter (SSC, 90° scatter) and were smaller in forward light scatter (FSC). This could be due to the behavior of the mitochondrial compartment. As expected, ShTaz1 cells had a lower SSC and FSC than ShWT1 cells and the ShTaz1R revertant cells (Table 1). This cellular hypertrophy of the ShTaz1 cells is also visible on the confocal microscopy pictures (Figure 6a,b).

#### 3.3.3. Seahorse Respiratory Measurements on intact Cells

Respirometry measurements using the Seahorse device on the three cell lines (ShWT1, ShTaz1 and ShTaz1R) showed that ShTaz1 cells had an elevated basal oxygen consumption rate (Figure 5c,d), increased F_1_F_0_ATPase synthase (Figure 5c,e), and increased H^+^ leak (Figure 5c,g), whereas their maximal respiratory capacity was widely compromised (Figure 5c,f).

Respiratory capacity, which reflects maximal electron transport chain activity, showed that TAZ deficiency and subsequently defective CL decreased the efficiency of ATP generation by lowering F1F0ATPase synthase activity.

The control cells (ShWT1 and ShTaz1R) had a respiratory capacity very close to the basal value (Figure 5c,d). Also similar to basal values were their proton leak (Figure 5g) and F_1_F_0_ATPase function (Figure 5e), whereas the maximal respiratory capacity of the ShTaz1R (revertant) never reached the value of the control cells (ShWT1) (Figure 5c,f), even if the difference could be considered as not significant.

### 3.4. Tafazzin Knockdown Activates AMPK and Increases Mitochondrial Density

The F_1_F_0_ATPase synthase activity was reduced as expected given the low amount of ATP synthesized in the ShTaz1 mutant (Figure 5d). This ATP comes from mitochondrial activity since glutamine must be metabolized by oxidative phosphorylation in the mitochondria to enable ATP generation [24]. The ATP produced in the ShWT1 control and ShTaz1R cells was almost similar. Thus, tafazzin knockdown lowers mitochondrial ATP production.

As described in Figure 6a,b and also evidenced in terms of light scatter (Table 1), the tafazzin knockdown mutant cells were larger than the ShWT1 control cells. This may be related to quite important metabolic changes.

A large increase in the pAMPK to AMPK ratio was recorded in the mutant cells (ShTaz1) (Figure 6d). These measurements should be interpreted in a context where the mitochondrial density (Figure 6e) is clearly increased in ShTaz1 cells (Figure 5b, EM picture), even if the mitochondria appeared smaller and fragmented (Figure 5b both pictures at right and left). Concerning the revertant cells (ShTaz1R), the cellular volume was not fully restored. Also, this cellular swelling (“cellular hypertrophy”) was associated with an increase in protein synthesis, as detected by 3[H]-leucine incorporation (Figure 6e). 

We also investigated whether tafazzin knockdown induces the hypertrophic marker gene and brain natriuretic peptide (BNP) expression. As shown in Figure 6f, tafazzin knockdown significantly increased the expression of BNP as determined by semiquantitative qPCR. The activation of BNP gene transcription was assayed by measuring a hBNP promoter coupled to a luciferase reporter gene. Tafazzin knockdown significantly increased BNP promoter activity compared with the scrambled virus control (Figure 6f). Thus, tafazzin knockdown clearly induced cellular hypertrophy in ShTaz1 cells.

When coupled to a luciferase reporter gene, the hBNP promoter that activated BNP gene transcription exhibited enhanced activity. Tafazzin knockdown significantly increased BNP promoter activity compared with the ShWT1 control or the ShTaz1R revertant cells (Figure 6g). In general terms, tafazzin knockdown induced hypertrophy plus all linked reporter events in HeLa cells.

When the mitochondria were smaller with reduced cristae membranes, they usually contained fewer respiratory complexes. This was analyzed in lymphocytes and myoblasts from the heart of BTHS patients. We investigated whether this affected mitochondrial function and oxidative phosphorylation. It was important to see whether the potential decrease in respiratory complexes and the recorded alteration in cristae structure affected mitochondrial bioenergetics, by determining ATP synthesis and mitochondrial respiration. 

The activity of citrate synthase, an enzyme of the mitochondrial matrix usually used to report on mitochondrial content, was clearly increased in ShTaz1 cells (Table 2) versus the control cells (ShWT1). This suggests that ShTaz1 has a greater mitochondrial content than the control and revertant cells (ShWT1 and ShTaz1R). As shown in Table 2, ATP synthesis was lower in the mutant cells (ShTaz1) than in the control and revertant cells.

### 3.5. Acidic Compartments and Autophagy Inhibition

**Figure 7:**
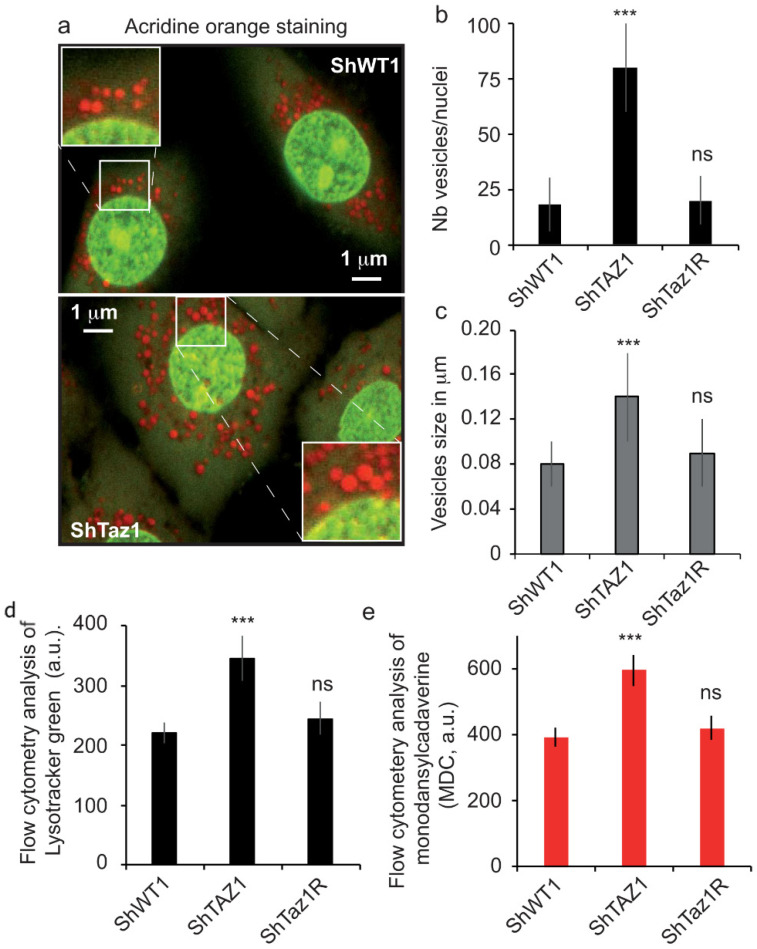
Confocal images and flow cytometry analysis of the acidic vesicles formed in the cytoplasm of control HeLa cells (ShWT1) and tafazzin knockdown cells (ShTaz1). (**a**–**c**) Analysis of the three HeLa cell lines (ShWT1, ShTaz1 and ShTaz1R) stained with AO. (**a**) Acridine orange staining of the lysosomes and other acidic compartments. At the top, ShWT1 control cells and bottom, ShTaz1 tafazzin knockdown. (**b**) Histogram representation of the number of red vesicles per nucleus. Calculation from 10–20 image field as presented in Figure 6a,b. (**c**) Histogram representation of vesicle size in μM calculated in the same image fields as presented in Figure 6a,b. (**d**) Flow cytometry analysis of the accumulation of LysoTracker Green in arbitrary units (a.u.). (**e**) Flow cytometry analysis of staining by monodansylcadaverine (MDC, in arbitrary units). ns = no significant and *** is the *p*-value ≤ 0.001.

As seen in Figure 6a,b, the cellular hypertrophy in ShWT1 cells linked to tafazzin knockdown cells was accompanied by an increase in AO red staining. Not only did the number of acidic vesicles per nucleus increase (Figure 7b), but their size was also significantly greater (Figure 7c). The acidic compartment returned to normal in the revertant cells (ShTaz1R) compared to the control cells (shWT1).

This increase in acidic vesicles allowed us to speculate about a possible increase when using other acidotropic markers like LysoTracker Green and MDC, which has also been reported to stain the autophagosomal membrane, thereby providing an extended definition of the type of vesicles stained [59,68].

The histogram profiles of LysoTracker Green staining (Figure 7e) and of MDC staining were very similar (Figure 7e), with a clear enhancement of staining with both probes in the ShTaz1 tafazzin mutant, staining in the ShTaz1R cells being very similar to that of the control cells. Since MDC is known to stain autophagosomes and lysosomes, this observation can complement the staining of lysosomes with LysoTracker Green (Figure 7d,e).

### 3.6. Abolition of Mitophagy in Tafazzin Cell Mutants

TAZ depletion appears to be the primary cause of the oxidative stress that is associated with impaired mitochondrial respiration. This is confirmed by our findings (Figure 5) on cellular respiration and ATP depletion (Figure 5d), which show that mutant cells have a lower basal respiratory capacity and that impaired cellular respiration takes place in a deleterious context of higher protein carbonylation (Figure 2b) and lipid oxidation (Figure 2c). Moreover, electron microscopy pictures showed that the mitochondria of tafazzin knockdown cells (ShTaz1) are altered, translucid, smaller, have fewer cristae membranes, and, most importantly, are still present in the cell cytoplasm. Usually, defective or dysfunctional mitochondria are discarded from cells by a very specific mechanism called mitophagy.

In order to follow what happens when tafazzin is knocked down, we used flow cytometric image analysis to monitor the acidic compartment with LysoTracker Green (or as an alternative MDC) and the fate of altered mitochondria, even if they have a slightly lower ΔΨm as assayed with TMRM. 

**Figure 8:**
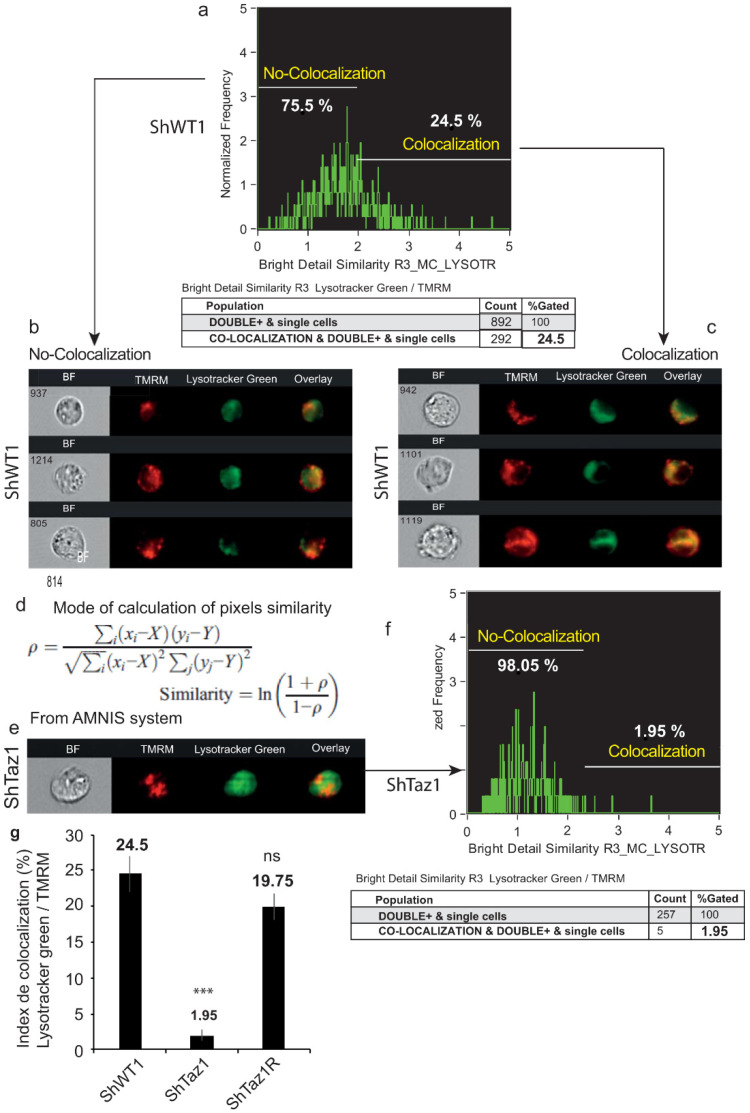
Image analysis of the co-localization of LysoTracker Green (lysosomes and acidic fusion vesicles) and the mitochondrial membrane potential dye, TMRM. (**a**) Colocalization based on brightness similarity and the corresponding histogram with 75.5% of the cells exhibiting different similarity indices, whereas 24.5% exhibit strict co-localization estimated by the similarity index. (**b**) Images of cells with similarity index < 2; no co-localization of the two probes. (**c**) Images of cells with similarity index > 2, which indicates co-localization of mitochondria in the acidic vesicles. (**d**) Method of calculation used to estimate the similarity index. (**e**,**f**) Analysis of ShTaz1 cells, which are deficient in tafazzin; (**e**) images and (**f**) curve of co-localization. (**g**) The co-localization index is reported on a histogram for the three cell types (ShWT1, ShTaz1, and ShTaz1R). ± SEM on 5 different preparations. ns = no significant and *** is the *p*-value ≤ 0.001.

In the control cells (ShWT1) where autophagy takes place, a large proportion of the mechanism is devoted to the more specific mitophagic events. Double staining with LysoTracker Green and the mitochondrial marker TMRM identified (Figure 8a–c) where these two markers are co-localized.

Mitophagy (defective mitochondria within specialized autophagosomes) was detectable in 24.5% of the cells (ShWT1 control). Calculation of the similarity index (Figure 8d) showed (Figure 8e–g) that there was almost no co-localization of the two probes in ShTaz1 cells (1.95% similarity) (Figure 8g), whereas the ShTaz1R revertant cells had a similarity index of 19.75% (Figure 8g), a value close to the 24.5% of the ShWT1 control cells. 

These results are very surprising since they clearly mean that the number and size of acidic vesicles were greatly increased, suggesting that not all autophagy mechanisms were altered and that there is a blockage of autophagic processes with a huge accumulation of acidic vesicles that are stained by LysoTracker Green, MDC, and also AO markers [67].

### 3.7. Changes of the Acidic Compartment in Normal and Rapamycin-Induced Autophagy

**Figure 9:**
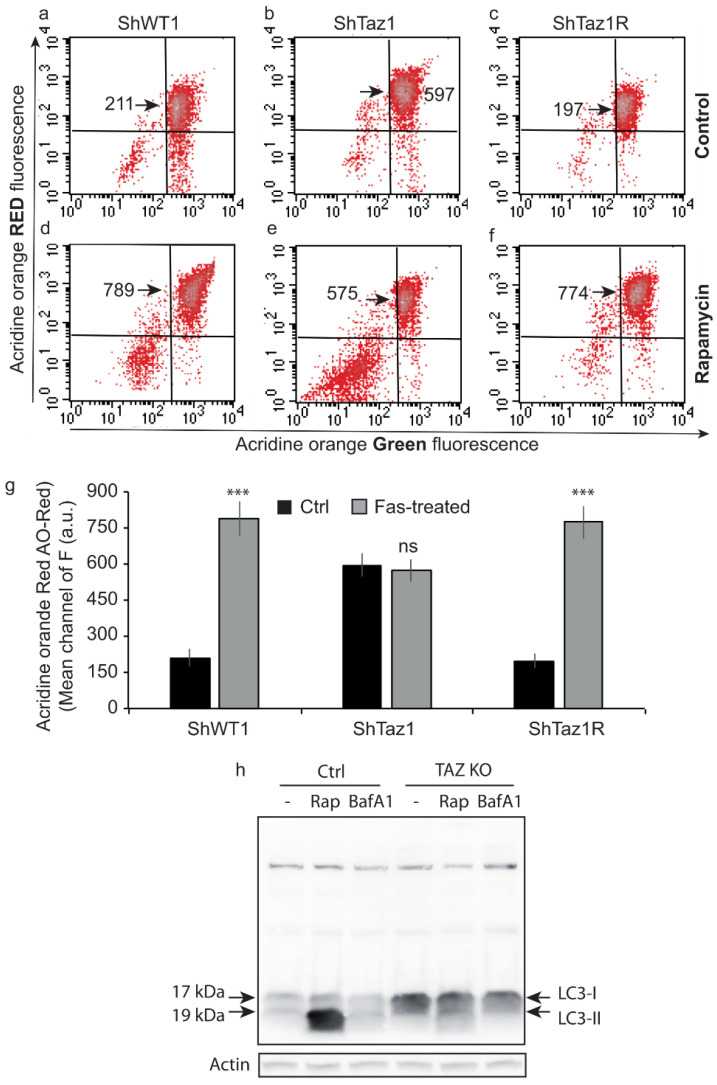
Flow cytometry analysis of acridine orange staining and LC3 Western blot. (**a**–**f**) Flow cytometric analysis of AO staining for the three cell lines (ShWT1, ShTaz1, and ShTaz1R) as described in materials and methods when treated or not with rapamycin for 24 h. Green fluorescence when the AO molecules are free or bind to the DNA; red fluorescence in an acidic environment where the molecules form stacks (Stokes shift). The histograms of green and red fluorescence show the enhancement of the acidic compartment (red fluorescence), but also the death of cells, which usually lose their acidic vesicles and have a compacted DNA resulting in less green fluorescence (green fluorescence). (**a**–**c**) Basal acidic vesicles/untreated cells (**d**–**f**) Cells treated with rapamycin (autophagy inducer) for 24 h. (**g**) Histogram representation of red fluorescence (mean values) in monitoring of acidic vesicle (lysosomes and certainly autophagosomes) statistics are given for 12 different preparations. (**h**) Western blot analysis of LC3 staining following basal, or rapamycin, or bafilomycin A3 treatment of the three cell lines (ShWT1, ShTaz1 and ShTaz1R). ns = no significant and *** is the *p*-value ≤ 0.001.

A detailed flow cytometric analysis of the green and red fluorescence of AO allowed us to follow the behavior of the three cell lines from the point of view of the current autophagic processes (i.e., essentially mitophagy) or of rapamycin-induced autophagy (Figure 9a–f) The mean cellular population was detected in green (principally by staining of nuclei, even if there was some green in the cytoplasm) and in red following the staining of the acidic vesicles (i.e., lysosomes and autophagolysosomes or mitophagosomes; the cells taken into account were TO-PRO-3-negative, which means viable).

The tafazzin-deficient cells (ShTaz1) (Figure 9b) had a more intense red fluorescence than the ShWT1 control (Figure 9a) or the ShTaz1R cells (Figure 8c). The mean fluorescent red values are presented in the histogram (Figure 9g). Less than 5% of cells in the lower red corner exhibit low green and low red fluorescence (Figure 9a–c).

When the cells were treated with 50 nM rapamycin for 12 h (to induce autophagy) they exhibited strange behavior (Figure 9d–f). Whereas, ShWT1 and ShTaz1R cells exhibited a large increase in AO red fluorescence (respectively, Figure 9d,f)—789 versus 211 in control cells (ShWT1) and 774 versus 197 in revertant cells (ShTaz1R) without such a large increase in the lower left corner (not more than 12%)—fluorescence in the ShTaz1 cells knockdown for tafazzin did not increase after treatment (Figure 9e). This means that the ShTaz1 cells did not respond to rapamycin and undergo strict inhibition of the further increase in the number and size of acidic vesicles and therefore in mitophagy. Significant also was the appearance of a large cellular population in the lower left corner (25–30%) that has low green and low red fluorescence, meaning that the DNA is more compacted and can no longer fix the AO molecules. And this tells us that the acidic compartment is altered (the acidic vesicles became permeable or were destroyed) and, as a consequence, is unable to accumulate the acidotropic dye. So, there are fewer stacked AO molecules and subsequently a lower red fluorescence (Figure 9e).

### 3.8. Assessment of Autophagic Processes with LC3-I and LC3-II

As a catabolic process, autophagy results in a situation where cellular contents including organelles are degraded using autophagic vacuoles [69]. Autophagy is a continuous process that occurs at low levels in normal cells and can be activated by various cellular stressors, including organelle damage or destabilization. Whereas in a situation where the cells are crucially dependent on defective mitochondria, as with ShTaz1 cells, quality control mechanisms that recognize and recycle damaged mitochondria should be critically regulated. Mitochondria quality control could be regulated by antioxidant and chaperone expression, localized remodeling/proteolysis, and autophagic degradation [70]. Concerning our present task, the recently discovered mitophagic pathway involving protein to lipid interaction and more precisely the interaction of LC3 with CL [42,71] is of great interest. Effectively, the mitophagic processes could be altered since CLs are in lower amounts and do not present the usual fatty acids involved in the interaction with LC3.

Western blot analysis of the control ShWT1 cells and the ShTaz1 mutant cells yielded complementary information (Figure 9h). The ShWT1 cells initially exhibited low amounts of the two forms of LC3-I and LC3-II. Rapamycin-induced autophagy of the ShWT1 cells is linked to the induction of a huge amount of LC3-II (Figure 9h, line 1). This was fully inhibited by 20 nM bafilomycin A3 (Figure 9h, line 2). The situation was quite different for the ShTaz1 tafazzin mutant cells, since the control cells exhibited a high amount of detectable LC3-I (Figure 9h, line 4), but rapamycin had no or little effect since it generated a very low level of LC3-II (Figure 9h, line 5), which could be abolished by bafilomycin A3 (Figure 9h, line 6). So, it appears that the large increase in acidic vesicles is not correlated with increased autophagy and, moreover, that the mitophagic processes are completely inhibited.

## 4. Discussion

We generated a model of BTHS in HeLa cells by RNA interference to obtain tafazzin knockdown. Using either a tafazzin-specific sequence or a non-specific Sh control, we constructed a short hairpin RNA (ShRNA)-encoding plasmid. HeLa cells were stably transfected and different clones were isolated (called ShTaz “n” or ShWT “n” control). During this study we used exclusively the ShTaz1 mutant, the ShTaz1R (revertant) and the ShWT1 control cells. Quantitative RT-PCR demonstrated that endogenous tafazzin RNA levels were significantly reduced in the ShTaz1 mutant (Figure 1a). Western blotting for endogenous tafazzin again proved the efficient knockdown of the protein (Figure 1b). We also performed CL and MLCL analysis and tafazzin knockdown cells presented lower amounts of CL, with significant changes in acyl chain length (as evidenced by a shift to the left of all CL species) (Figure 1c top and bottom) and a small but significant increase of MLCL. Whereas the increase of MLCL is modest in the ShTaz1 HeLa cell mutant compared with BTHS-derived lymphoblastoid cells [37,50] and with BTHS iPSC-derived cardiomyocytes [72], a significant and comparable decrease in CL is evident in the ShTaz1 mutant, with a dramatic loss of the major peak of each cluster of CL species (Figure 1c). As in BTHS patients [73], the lack of tafazzin in HeLa cells has almost no effect on other major phospholipids (Figure 1d). For further use, we introduced a mutation into the cDNA of human tafazzin, and ShTaz1 mutant cells were transfected with this cDNA to generate stable revertant clones (among which we chose to work specifically on the ShTaz1R clone). ShTaz1R cells express tafazzin to a level mainly comparable to the control HeLa ShWT1 cells (Figure 1b).

### 4.1. Inhibition of the Extrinsic Apoptotic Pathway in Tafazzin-Deficient Cells

CL-deficient HeLa cells (ShTaz1 knockdown cells) appear to be resistant to the induction of cell death by anti-Fas antibody (Figure 2), provided they do not exhibit any drop in ΔΨm as sensitive cells do (i.e., ShWT1 and ShTaz1R HeLa cells). These results are in strict accord with previous reports on other tafazzin-deficient cell lines (i.e., lymphoblasts or iPSC-derived cardiomyocytes), and focused our attention on the major role of mature CL in Fas-induced apoptosis. Clearly, these results again prove that CL-deficient cells are defective in terms of the extrinsic apoptotic pathway, which links the death receptor (Fas receptor) with the core pro-apoptotic machinery.

These important points have been described previously [37,50], but here we were also able to see that this inhibition of cell death signal transduction takes place in a cellular environment of high reducing conditions: Protein oxidation (Figure 2b) and lipid peroxidation (Figure 2c) are enhanced in the ShTaz1 tafazzin mutant. Concerning the lipids, a similar form of oxidative stress has been evidenced recently by Le et al. [74].

Additionally, we sought to identify where in the early events of apoptosis the blockage of apoptosis signaling takes place. Usually, ShWT1 and ShTaz1R cells, which are fully sensitive to anti-Fas antibody-induced apoptosis, exhibit the canonical early mitochondrially linked events as a drop in ΔΨm, a reduction in NADPH and NADH, enhanced superoxide anion production, and an activation of caspase-3/7 due to the release of cytochrome *c* [75,76,77,78].

Analysis of the apoptosis-associated events proved that, in ShTaz1 mutant cells, the characteristics of the cells are not fully restored (Figure 3a–d). Clearly, apoptosis is inhibited at the mitochondrial level and this is linked to the absence of mature CL at the mitochondrial membrane contact sites that interact with proteins acting as apoptosis effectors, i.e., caspase-8 or Bid molecules [37,50,79,80].

### 4.2. Alterations and Characteristics of Tafazzin-Knockdown HeLa Cell Proliferation

Impedancemetry is a useful tool for monitoring cellular proliferation [53] and yielded unexpected results. The three cell lines studied (ShWT1, ShTaz1, and ShTaz1R) exhibited quite different behavior in terms of all the characteristics accessible by impedancemetry. Both cell adhesion and proliferation curves were affected and differed from each other (Figure 4a). ShWT1 control cells exhibited a classical proliferation curve, with an adhesion sequence ending in a transition plateau before the proliferation started, then the maximum CI was reached and the CI started to decrease following cell death in culture (cells detached from the bottom of the wells). As expected, the ShTaz1 tafazzin-deficient cells were affected in all the steps, and had lower adhesion capacity as well as a reduced rate of proliferation and a lower maximum CI (Figure 4a). The technique is sensitive enough to prove that the revertant cells (ShTaz1R) do not have a fully restored capacity to bond and to proliferate, though they are more similar to the control cells (ShWT1). 

### 4.3. Bioenergetic Defects Lie in Mitochondrial Structure and Mitochondrial Homeostasis Destabilization

Electron microscopy analysis of ShTaz1 and ShWT1 cells showed that the ShTaz1 cells exhibited numerous small abnormal mitochondria (Figure 5). They had a translucid matrix and few cristae membranes. Their abundance seems to prove that they are not eliminated by specific mitophagy events, since few autophagosomes were found in the electron micrographs. Careful examination revealed no trace of defective mitochondria in specific phagosomal structures.

Measurement of cellular ATP production proved that ShTaz1 mutant cells have less ATP available to sustain cellular adhesion and proliferation, whereas ShTaz1R cells have an almost restored ATP production (Figure 6c) and that this is valuable whatever the conditions of ATP measurements are (in galactose where the glycolysis is favored or in glucose) (Table 3).

Clearly tafazzin knockdown causes hypertrophy in HeLa cells (Figure 6a–b). Decreased tafazzin expression lowers CL and modifies CL acyl chain composition in ShTaz1 mutant cells, and this in turn blunts ATP production by the mitochondria. 

ATP depletion has well-known detrimental effects that include cellular hypertrophy and may initiate cell death. Acute ATP depletion switches on the catabolic pathways (still producing ATP by use of fatty acid oxidation and glycolysis) and switches off the ATP-consuming anabolic pathways such as lipogenesis and gluconeogenesis [81]. With rare diseases like BTHS, it is always interesting to estimate the long-term effects of any metabolic modification. One long-term response to ATP depletion is enhanced mitochondrial biogenesis (Figure 6f,g), which is mediated by the activation of the “fuel gauge” AMPK (Figure 6d) [82]. Jager et al. [83] have shown that pAMPK directly phosphorylates and activates PGC1α, the key regulator of mitochondrial biogenesis [84].

We used an extracellular flux analyzer to probe mitochondrial functions (Figure 5c–h). The HeLa cells exhibited an unexpected high basal oxygen consumption rate which fitted perfectly with both an increase in F_0_F_1_ATP synthase and an increase in H^+^ leak. Since there is a reduction of the basal ATP levels, these results pointed out the inefficiency of the ATP synthase activity. These results proved that TAZ deficiency and associated CL abnormalities reduce electron transport chain function and decrease ATP generating system efficiency by lowering F_0_F_1_ATP synthase specific activity. 

### 4.4. Mitophagy Inhibition Due to Defective Cardiolipin in the Tafazzin-Deficient HeLa Cell Mutant

Mitophagy, the specialized autophagy that targets defective mitochondria, is a cardinal feature of mitochondrial quality control since it is responsible for the clearance of damaged mitochondria. Defective mitophagy is usually linked to the impairment of mitochondrial functions [85,86]. 

It well-known that mitochondria are flexible and highly dynamic structures. Mitochondrial division and fusion occurring in a coordinated cycle of “mitochondrial dynamics” [87] provide rapid adaptation of mitochondrial morphology and play an important role in the regulation of the cell cycle, cellular immunity, apoptosis, and mitochondrial mass [88]. Inadequate ATP supply and/or high production of ROS may promote dysfunction of mitochondrial dynamics that can directly damage cells. It is interesting to note that many human diseases are associated with mutations in mitochondrial core mechanical components and flaws in mitochondrial dynamics [89,90].

Mitochondrial dynamics is the process of mitochondrial fusion and fission, and determines the shape, quality and quantity of mitochondria [91,92]. Mitochondrial dynamics is closely linked to mitochondrial functions, such as cell proliferation, cell metabolism, and cell migration [92], and is tightly regulated by a variety of proteins.

### 4.5. Mitochondrial Dysfunctions are Compensated at the Cellular Level

Despite the disruption of mitochondrial homeostasis due to tafazzin deficiency, and the fact that this is accompanied by ATP depletion (Figure 5d and Table 3) and ROS generation that originate from the altered electron transport chain, there is a clear development of the acidic vesicular system that comprises lysosomal and late endosomes (Figure 7). But, even if the network of vesicles is clearly prone to mitophagy, the absence of mature CL with an appropriate acyl chain composition leads to its inhibition (Figure 8). So, dysfunctional mitochondria resulting from fission of the mitochondrial network are not eliminated and accumulate (Figure 5a,b). This results in damaged mitochondria, which otherwise would have been eliminated, remaining within the ShTaz1 cells.

Thus, although ShTaz1 cells exhibit lower levels of CL with fatty acid chain modifications, they can, nonetheless, fulfill cellular energy demands by a mechanism of compensation. This includes an increase in mitochondrial mass (despite smaller mitochondria), as shown by electron microscopy (Figure 5), as well as a significant increase in citrate synthase activity, which is an indication of an efficient de novo mitochondrial synthesis (Table 2 and Figure 5b).

### 4.6. Accumulation of Acidic Vesicles and Inhibition of Mitophagy

While altered and exhibiting a lower mitochondrial membrane potential, mitochondria are usually destined for degradation mediated by the PINK1- and PARK2-dependent pathway [93]; mitochondria with low ΔΨm and significant ROS production stayed present within the cells (Figure 8). Moreover, classical Western blotting of LC3 indicates that LC3-II is not generated in tafazzin-deficient cells (ShTaz1) (Figure 9), proving if necessary that mitophagy is not effective in these tafazzin mutant cells. These results are quite consistent with the recent results of Wang’s team on cardiomyocytes [72] that proved that Barth syndrome patients have deficient O_2_ consumption in muscle and also impaired cardiac capacity, which fit well with the observed exercise intolerance [73]. Our results also complement the indication provided by Hsu’s team that efficient CL remodeling is selectively necessary for proper mitophagy initiation [94].

In summary, our findings provide new insight into the pathogenesis of Barth syndrome, by highlighting the role played by aberrant CL, as tafazzin mutation leads to an impairment of tafazzin function and the production of smaller amounts of CL with modified acyl chains and the accumulation of MLCL. All this results in mitochondrial dysfunction. CL is a key element of electron transport chain efficiency and also a key structural component of mitochondrial membranes (and contact sites between the two membranes). One confirmed consequence is impairment of the pro-apoptotic signal that mediates the extrinsic cell death pathway (where the mitochondria act as an amplifier). This pathway is strictly abolished due to the inability of the CL-caspase-8-Bid platform to be formed [50,51,95]. Here, it appears that there is also a clear inhibition of the mitophagic events that maintain the less efficient but abundant mitochondria in the cytoplasm. This seems to work by almost the same mode of action since there is a lack of CL to LC3 interaction that abolishes mitophagy.

These results also highlight mitochondrial bioenergetic failure even in the presence of compensation at the cellular level. One major question for future studies concerns possible interventions, in three main directions: Targeting the oxidative status of these cells (by mitochondrially located antioxidants); intervention at the mitophagic processes; and/or possible restoration of the translation of the mitochondrial precursors through the mitochondrial membranes (assuming they are altered by the changes in mitochondrial membrane properties due to the alteration of CL).

## Figures and Tables

**Table 1:** Flow cytometry measurements of the cell light scatter.

Cell Type	Side Scatter (90° Light Scatter)	Forward Low-Angle Light Scatter (10–21° Angle)
ShWT1	257 ± 35	476 ± 18
ShTaz1	207 ± 29	442 ± 12
ShTaz1R	230 ± 31	458 ± 15

The ± values are from the flow cytometry histograms and represent the standard deviation to the mean fluorescent value at half-peak on the Gaussian representation calculated for 10,000 events.

**Table 2:** Mitochondrial activities measured in intact permeabilized HeLa cell lines (ShWT1, ShTaz1, and ShTaz1R).

Activities. nmol/min/mg	ATP Synthesis Activity	Citrate Synthase Activity	ATP/CS
ShWT1	25.8 ± 1.5	182.3 ± 24.6	0.141
ShTaz1	17.4 ± 1.2 *	287.5 ± 9.0 *	0.060 *
ShTaz1R	23.9 ± 1.4	201.6 ± 19.3	0.118
	**Coupled Respiration**	**Uncoupled Respiration**	**CS/Coupled**
ShWT1	7.7 ± 0.6	19.7 ± 0.8	0.042 ± 0.004
ShTaz1	6.9 ± 0.9	19.6 ± 1.8	0.024 ± 0.006 *
ShTaz1R	7.5 ± 0.6	19.8 ± 1.3	0.037 ± 0.007

ATP synthesis activity and citrate synthase activity (CS). The values shown are means of at least three independent measurements expressed in nmol/min/mg protein. Statistically significant differences between ShWT1 control and ShTaz1 or HeLaTaz1R cells are indicated: * *p* < 0.05.

**Table 3:** Measurement of the cellular ATP content in different culture conditions. Glycolysis is favored by cell culture in galactose, whereas both mitochondria and glycolysis are at work when the culture is on glucose.

ATP (pmole/mg protein)	Cell Culture on Galactose	Cell Culture on Glucose
ShWT1	61 ± 4	58 ± 8
ShTaz1	21 ± 5	48 ± 5
ShTazR1	55 ± 7	56 ± 6

## Abbreviations

AO: acridine orange; BafA1, bafilomycin A1; BTHS, Barth syndrome; mClCCP, carbonyl cyanide m-chlorophenylhydrazone; CL, cardiolipin; FCCP, carbonyl cyanide p-trifluoromethoxyphenylhydrazone; hBNP, human brain natriuretic peptide; LTG, LysoTracker Green; MAP1LC3B/LC3B, microtubule-associated protein 1 light chain 3 beta; mClCCCP, carbonylcyanide m-chlorophenylhydrazone; MDC, monodansylcadaverine; MLCL, monolysocardiolipin; ND1; NADH dehydrogenase 1 (mitochondrial); TMRM, MitoTracker Red; Rapa, rapamycin; ShWT1, HeLa cells, control empty vector; ShTaz1, HeLa cells, tafazzin knockout; ShTaz1R, HeLa cells, revertant; Tazz, tafazzin.
